# Sclerosing angiomatoid nodular transformation of the spleen (SANT) in a patient with clear cell carcinoma of the uterus: a case report

**DOI:** 10.1186/s13256-018-1907-5

**Published:** 2018-12-23

**Authors:** Boubacar Efared, Ibrahim S. Sidibé, Fatimazahra Erregad, Nawal Hammas, Laila Chbani, Hinde El Fatemi

**Affiliations:** 1 Department of Pathology, Hassan II University Hospital, Fès, Morocco; 2 Laboratory of Biomedical and Translational Research, Faculty of Medicine and Pharmacology, Sidi Mohamed Ben Abdellah University, Fès, Morocco

**Keywords:** Spleen, Sclerosing angiomatoid nodular transformation of the spleen (SANT), Clear cell carcinoma, Uterus, Pathology

## Abstract

**Background:**

Sclerosing angiomatoid nodular transformation of the spleen is a very rare benign vascular lesion recently described. Usually, sclerosing angiomatoid nodular transformation of the spleen is an incidental finding; the association with malignant tumors is extremely rare. To the best of our knowledge, we report the first case of sclerosing angiomatoid nodular transformation of the spleen associated with uterine clear cell carcinoma.

**Case presentation:**

A 49-year-old Arabic woman presented to our institute with abdominal pain and distention. An abdominal computed tomographic scan was obtained, which showed a 14-cm uterine malignant tumor and a 4-cm isolated splenic nodule suggesting a metastatic lesion. The tumor was limited to the uterus but did not extend beyond. The patient underwent surgical treatment, and the histopathological examination of the resected uterine and splenic specimens disclosed invasive uterine clear cell carcinoma and sclerosing angiomatoid nodular transformation of the spleen, respectively. The patient had no signs of the disease 17 months after surgical treatment.

**Conclusions:**

Sclerosing angiomatoid nodular transformation of the spleen is a very rare benign disease with a misleading presentation when associated with a malignant tumor. Pathological assessment of the resected spleen is the only way to achieve the correct diagnosis.

## Background

Sclerosing angiomatoid nodular transformation of the spleen (SANT) is a very rare benign vascular lesion recently described by Martel *et al.* [1]. Since then, at least 135 cases have been reported [2]. The pathogenesis of SANT remains a controversial issue. Some authors consider it as a reactive inflammatory disease linked to immunoglobulin G4 (IgG4)-related sclerosing disease rather than as a true neoplastic process [1, 3–5]. More recently, Chang *et al*. found that SANT is a polyclonal reactive lesion instead of a neoplastic disease [6]. Mostly, SANT has been reported as an incidental finding, rarely found in symptomatic patients [1, 2, 6]. Rare cases of SANT have been reported in patients with malignant tumors, and the diagnosis of splenic metastasis has been raised because imaging techniques lack any specificity in the diagnosis of SANT [2, 7–9]. The histopathological analysis of splenectomy specimens is the only way to achieve a correct diagnosis [10, 11].

We report an additional case of SANT associated with uterine clear cell carcinoma in a 49-year old woman. To the best of our knowledge, this is the first case of SANT diagnosed in a patient with concomitant uterine clear cell carcinoma. The current literature offers only some case reports and short series about SANT, and unfortunately the management of this disorder is still not well defined. We think that accumulation of additional cases will bring more information about the disease and that therefore both clinicians and pathologists would become more familiar with it, thus preventing misdiagnosis and mismanagement, especially unnecessary splenectomy.

## Case presentation

A 49-year old Arabic housewife presented at our hospital for abdominal pain and distention. Her medical history was unremarkable apart from uterine myomectomy 17 years earlier. The patient did not smoke or drink alcohol. On admission, she had no fever, her body temperature was 37.5° C, her pulse rate was 70 beats/minute, and her blood pressure was 120/80 mmHg. The result of her neurological examination was normal. Her laboratory test results were within normal limits (especially the complete blood count and liver and renal function).

The patient’s physical examination showed a distended abdomen with a large tumor extending from the pelvis to the umbilical area. An abdominal computed tomographic (CT) scan was obtained, which showed a 14-cm uterine malignant tumor. Also, the CT scan revealed a 4-cm isolated splenic nodule suggesting a metastatic lesion. The tumor was limited to the uterus and did not extend beyond.

**Fig. 1:**
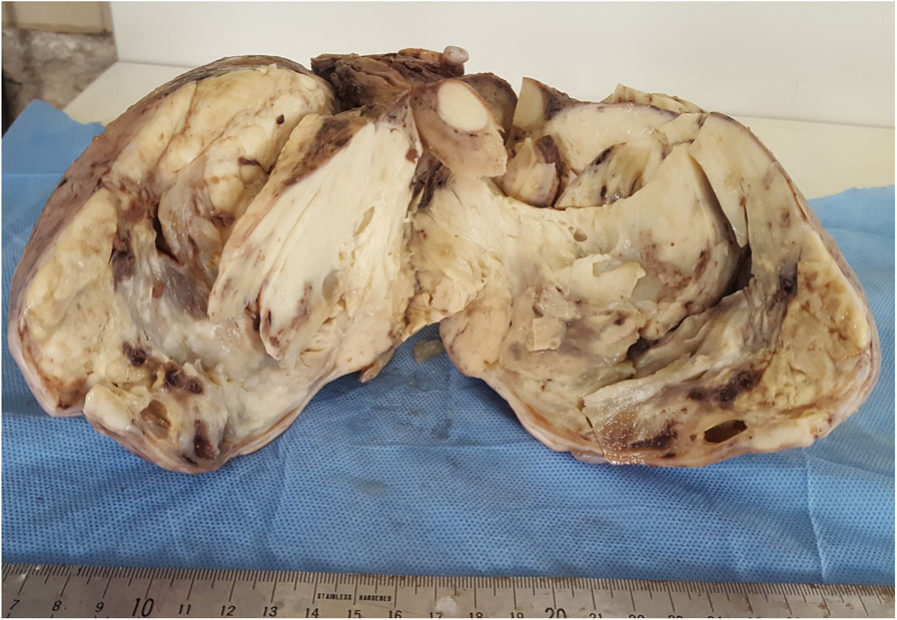
The resected uterus is entirely occupied by a whitish tumor with areas of cystic changes

**Fig. 2:**
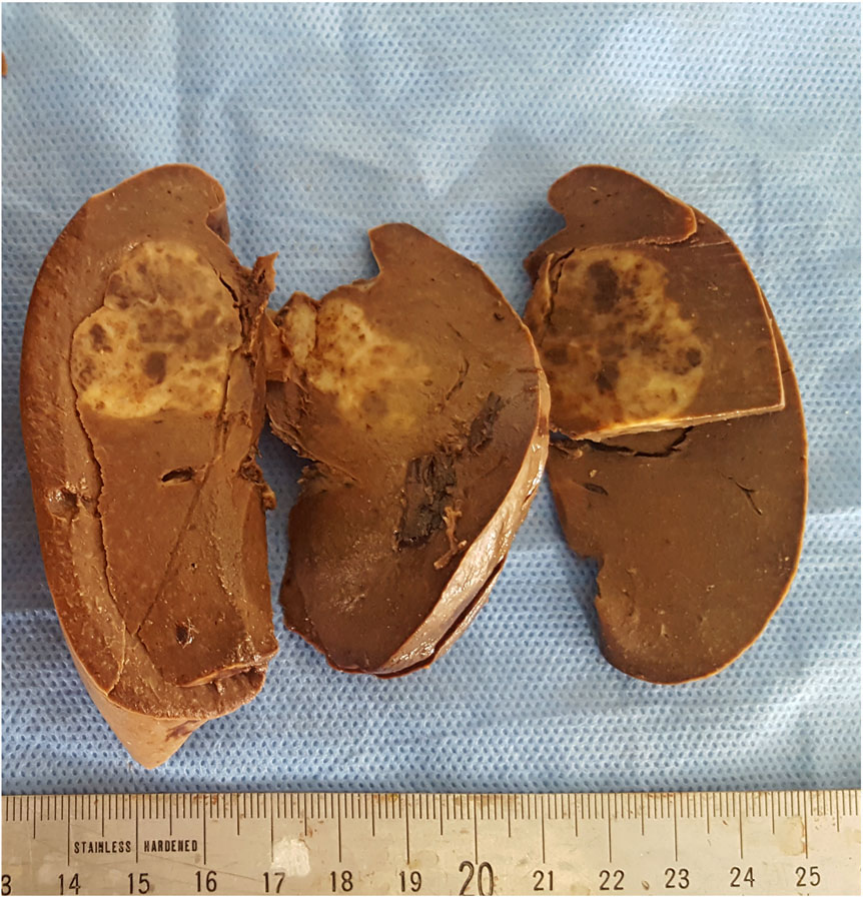
The spleen shows an ill-defined heterogeneous lesion with irregular contours

**Fig. 3:**
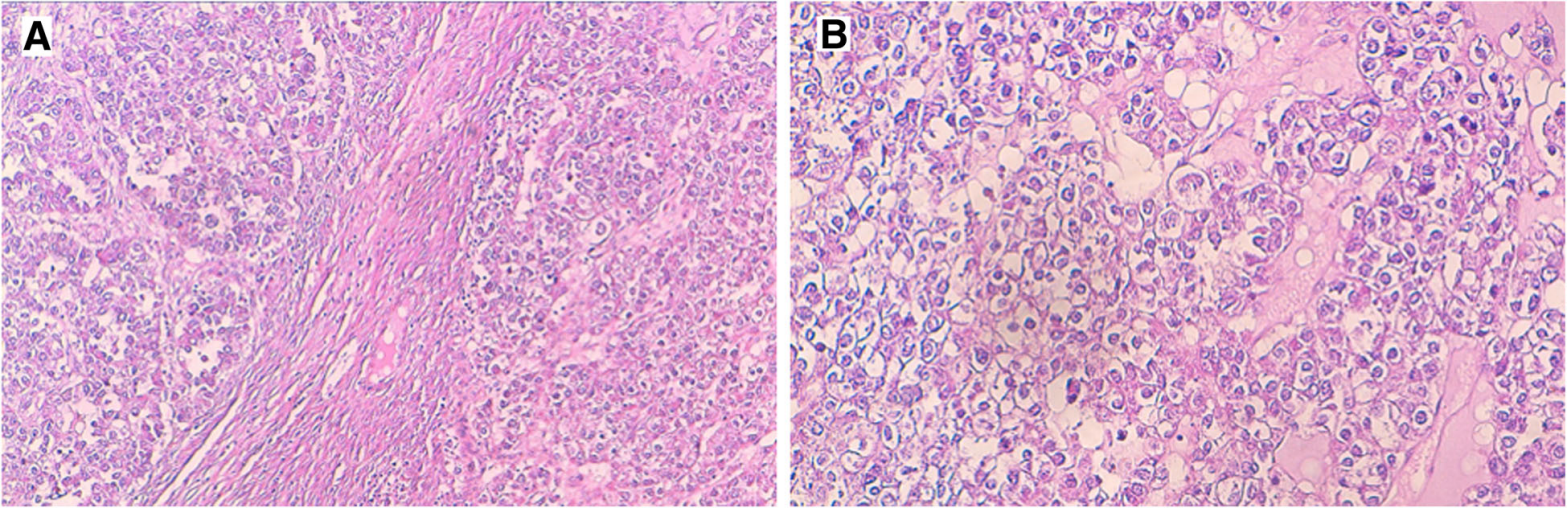
**a** Uterine clear cell carcinoma with solid and trabecular structures invading the myometrium (H&E stain, original magnification × 50) **b** Tumor cells have abundant granular clear cytoplasm with rounded nuclei (H&E stain, original magnification × 200)

The patient underwent radical surgical treatment consisting of total hysterectomy, total splenectomy, and lymphadenectomy. The macroscopic examination of resected specimens showed a huge, whitish, friable tumor occupying the entire uterine cavity, as well as a 4-cm splenic nodule with irregular contours, fibrous consistency, and a heterogeneous aspect (Figs. 1 and 2, respectively). The histopathological analysis disclosed a uterine clear cell carcinoma invading the outer half of the myometrium with negative lymph nodes, classified as stage IB according to the International Federation of Gynecology and Obstetrics classification scheme (Fig. 3a and b). The tumor cells were disposed in irregular trabecular and solid structures with abundant granular amphophilic to clear cytoplasm and rounded nuclei. The tumor cells were negative for estrogen and progesterone receptors and positive for cytokeratin AE1/AE3.

**Fig. 4:**
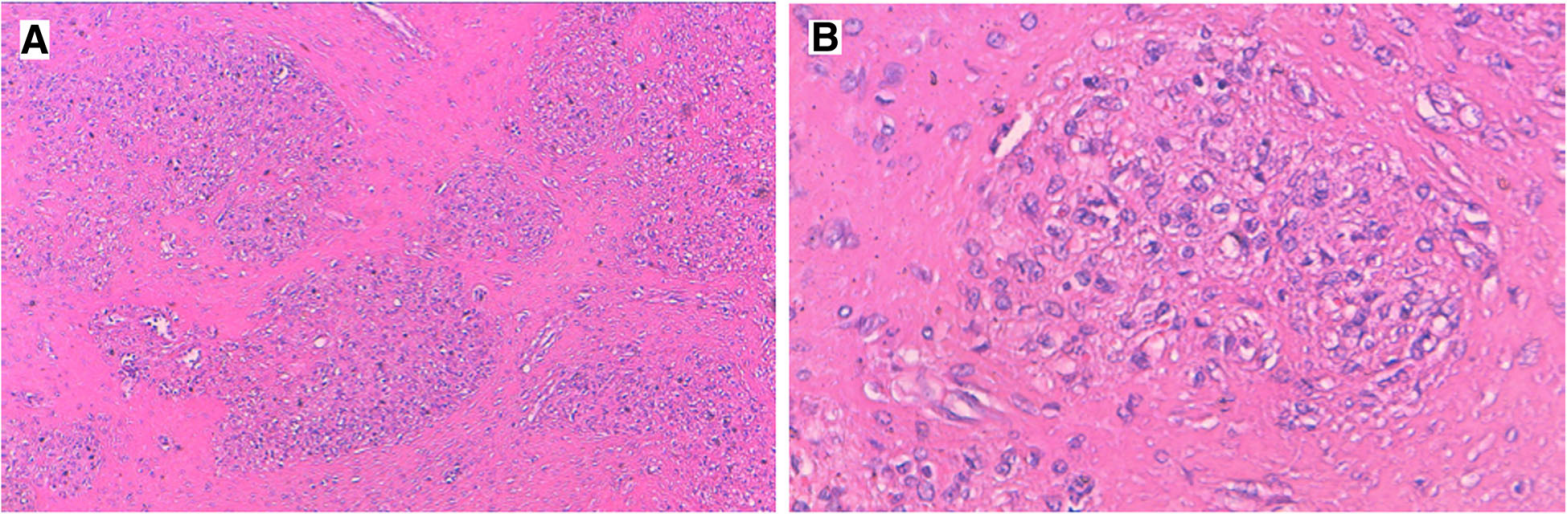
**a** The histological image of sclerosing angiomatoid nodular transformation of the spleen shows fibrotic bundles surrounding variably sized angiomatoid nodules (H&E stain, original magnification × 100). **b** Angiomatoid nodule with small vascular spaces layered by regular endothelial cells (H&E stain, original magnification × 200)

**Fig. 5:**
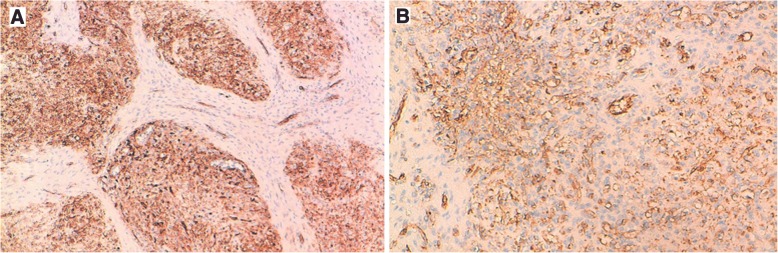
**a** Some vessels of the nodules are positive for CD34. **b** Positive immunostaining for CD31 in some vessels (IHC stain, original magnification × 100 and × 200, respectively)

**Fig. 6:**
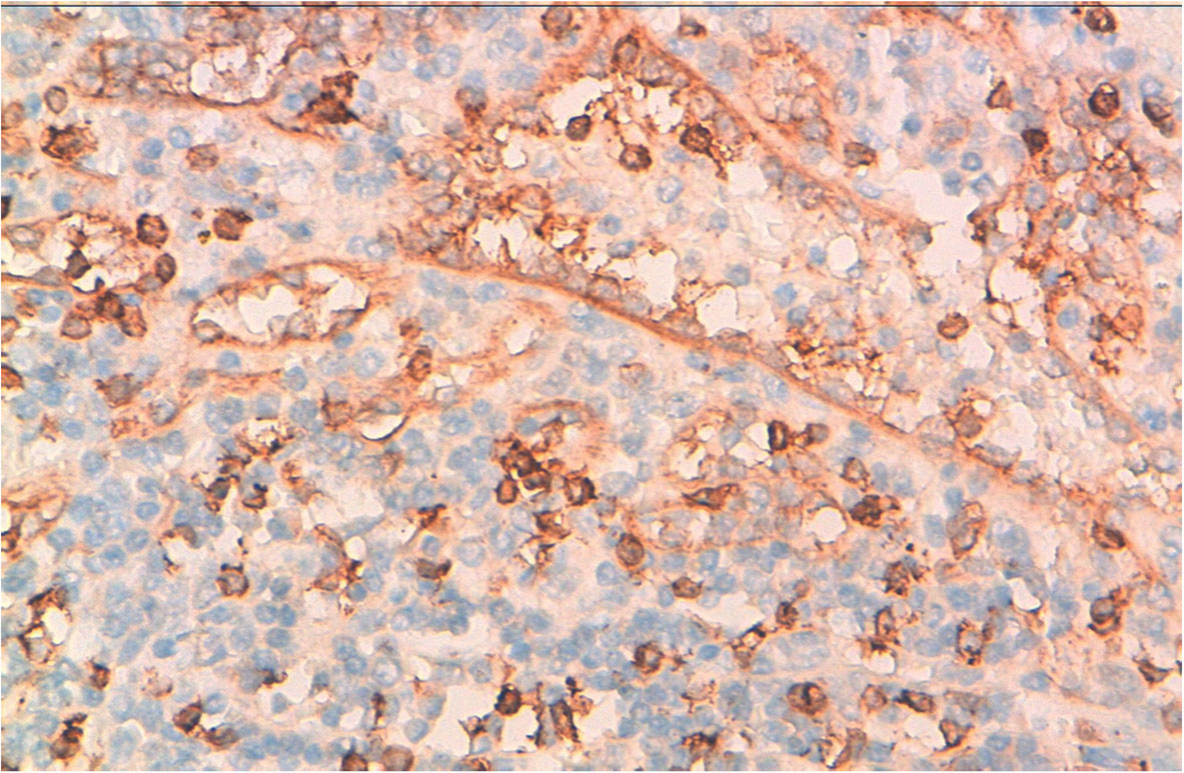
Within the nodules, some vessels are positive for CD8. Scattered CD8^+^ lymphocytes are also present (IHC stain, original magnification × 400)

The splenic lesion consisted of multiple confluent and variably sized fibrotic nodules centered by many vessels. These vessels were surrounded by areas of hemorrhage, fibroblasts, and hemosiderin-laden macrophages (Fig. 4a and b). The immunohistochemical analysis of the splenic lesion showed that these vessels had three distinctive immunophenotypes corresponding to splenic red pulp vessels: CD34^+^/CD8^−^/CD31^+^, CD34^−^/CD8^−^/CD31^+^, and CD34^−^/CD8^+^/CD31^+^, respectively (Figs. 5a, b and 6). These histopathologic features were consistent with SANT. The patient had no signs of the disease 17 months after the surgical treatment.

## Discussion

We report a case of a patient with SANT associated with uterine clear cell carcinoma that was radiologically misdiagnosed as a splenic metastasis. Association between SANT and malignant tumors is extremely rare. In our patient, splenic metastases were always suspected, and as a result, she underwent unnecessary splenectomy. Unfortunately, preoperative diagnosis was not possible, and the correct diagnosis was reached only by histopathological examination of the resected spleen.

SANT is a newly described pathologic lesion. It was initially reported in 2004 by Martel *et al.* in 25 cases of splenic lesions [1]. Since 2004, approximately 135 cases have been reported in the literature [2]. It is well accepted that SANT has a benign clinical course; however, its reactive or neoplastic nature remains controversial [1, 3–5]. It has been suggested that SANT is associated with IgG4 sclerosing lesions or reactive inflammatory pseudotumors [3, 4]. Recently, Chang *et al*. found that SANT is a polyclonal lesion and suggested consideration of that entity as a reactive lesion rather than as a genuine neoplasm [6]. Most reported cases of SANT were female patients, and usually they were diagnosed on the basis of incidental findings obtained using imaging techniques [9]. These techniques do not show specific features in SANT. Sometimes they are even misleading when patients present with malignant tumors [2, 7, 8]. In these instances, as in our patient, radiological techniques suggest the diagnosis of splenic secondary tumor leading to unnecessary surgical treatment [2]. Regarding imaging techniques, some authors have suggested that a “spoke wheel pattern” is a valuable diagnostic clue for SANT [9]. Lewis *et al.* showed that typical radiological features of SANT are a solitary, round, lobulated mass with early peripheral enhancing radiating lines and progressive enhancement of the angiomatous nodules, delayed enhancement of the fibrous tissue, and hypointense T2 signal intensity from hemosiderin deposition [12]. On positron emission tomographic (PET) scans, lesions with low ^18^F-fluorodeoxyglucose (FDG) accumulation should be considered as SANT, in addition to other vascular tumors [13].

In the literature, cases of SANT have been reported in patients with chronic lymphocytic leukemia, squamous cell carcinoma of the lung, colonic carcinoma, gastric carcinoma, chromophobe renal cell carcinoma, malignant melanoma, urothelial carcinoma, basal cell carcinoma, and spindle cell sarcoma [1, 2, 9]. Also, SANT has been reported in association with other nonneoplastic conditions such as pregnancy, von Willebrand disease, hypothyroidism, and chronic hepatitis [9, 10]. The pathophysiology of SANT or its association with other diseases, whether malignant or not, is not clearly defined. It has been speculated that SANT develops from splenic vascular insult, and a subsequent healing reaction occurs with vascular proliferation [1].

To the best of our knowledge, our current case report describes the first case of SANT that was associated with a concurrent uterine clear cell carcinoma. Because preoperative diagnosis of SANT is challenging, the histopathological analysis of the resected splenic specimen remains a unique way to achieve a correct diagnosis. Grossly, SANT usually appears as a poorly limited nodule with a radiating central fibrous scar with many embedded small brown nodules [1, 6]. Some cases of multifocal SANT have been reported [14]. In histopathological analysis, SANT recapitulates approximately a splenic red pulp dissected by fibrotic bundles forming variably sized nodules [1]. Within the nodules, three distinct vascular structures are found, consisting of capillaries (CD34^+^/CD8^−^/CD31^+^), splenic sinusoids (CD34^−^/CD8^+^/CD31^+^), and small veins (CD34^−^/CD8^−^/CD31^+^) [6]. The internodular fibrotic stroma contains inflammatory cells comprising IgG4^+^ plasma cells, extravasated erythrocytes, and CD68^+^ histiocytes. Cases of SANT positive for CD30 or Epstein-Barr virus-encoded small RNA have been reported in the literature [6, 15]. In fact, SANT has distinctive characteristics that allow ruling out of the diagnosis of other splenic vascular lesions. Hemangioma and littoral cell angioma are the main benign splenic vascular tumors [1, 6]. In contrast to SANT, these lesions are monophasic vascular tumors and lack the nodular fibrotic architecture. Hemangioma shows usually large vascular spaces rather than discrete vascular lumens seen in SANT. Also, the lack of cellular atypia and a low mitotic proliferative index in SANT easily rule out the diagnosis of malignant splenic vascular tumors such as hemangioendothelioma or angiosarcoma.

The management of patients with SANT is unfortunately not well defined, because preoperative diagnosis is impossible. In patients with concomitant malignant tumors, the occurrence of SANT is often mistakenly interpreted as a secondary splenic tumor leading to unnecessary splenectomy, despite the rarity of splenic metastases [2, 7, 10]. Ideally, asymptomatic patients with SANT should not undergo splenectomy. However, the challenging issue remains the preoperative diagnosis of SANT in order to prevent asymptomatic patients from unnecessary surgery.

## Conclusions

Our patient’s case of SANT associated with a malignant tumor emphasizes the lack of effective preoperative diagnostic tools that are able to detect the benign clinical nature of the lesion. Such diagnostic tools would prevent patients from unnecessary splenectomy. The histopathological assessment of the resected spleen remains the only way to achieve the correct diagnosis.

## Abbreviations

CT: Computed tomographic
FDG: ^18^F-fluorodeoxyglucose
IgG4: Immunoglobulin G4
PET: Positron emission tomographic
SANT: Sclerosing angiomatoid nodular transformation of the spleen
